# Analysis of Different Approaches for the Selection of Reference Genes in RT-qPCR Experiments: A Case Study in Skeletal Muscle of Growing Mice

**DOI:** 10.3390/ijms18051060

**Published:** 2017-05-16

**Authors:** Verónica G. Piazza, Andrzej Bartke, Johanna G. Miquet, Ana I. Sotelo

**Affiliations:** 1 Universidad de Buenos Aires, Consejo Nacional de Investigaciones Científicas y Técnicas, Instituto de Química y Fisicoquímica Biológicas (IQUIFIB), Facultad de Farmacia y Bioquímica, Buenos Aires C1113AAD, Argentina; vpiazza@qb.ffyb.uba.ar (V.G.P.); jmiquet@qb.ffyb.uba.ar (J.G.M.); 2 Department of Internal Medicine and Physiology, Division of Geriatric Research, School of Medicine, Southern Illinois University, Springfield, IL 62794-9628, USA; abartke@siumed.edu

**Keywords:** reverse transcription-quantitative PCR, normalization strategy, reference genes, total RNA, isolated messenger RNA, dilution effect, growth hormone, skeletal muscle, growing mice, signal transduction

## Abstract

The reliability of reverse transcription-quantitative PCR (RT-qPCR) results in gene expression studies depends on the approaches used to account for non-biological variations. In order to find a proper normalization strategy for the study of genes related to growth hormone signaling in skeletal muscle of growing mice, nine unrelated genes were evaluated as internal controls. According to the most used algorithms–geNorm, the Comparative Δ*C*q method, NormFinder and BestKeeper–*GSK3B*, *YWHAZ*, *RPL13A* and *RN18S* were found as the most stable. However, the relative expression levels of eight of the potential reference genes assessed decreased with age in cDNA samples obtained from the same amount of total RNA. In a different approach to analyze this apparent discrepancy, experiments were performed with cDNA obtained from equal amounts of purified mRNA. Since the decline was still observed, the hypothesis of an age-related change in mRNA to total RNA ratio that could account for the systematic decrease was rejected. Differences among experimental groups could be due to a substantial increase with age in highly expressed mRNAs, which would bias the quantitation of the remaining genes. Consequently, those reference genes reflecting this dilution effect, which would have been discarded considering their variable relative expression levels, arose as suitable internal controls.

## 1. Introduction

Reverse transcription-quantitative PCR (RT-qPCR) is the most widely used technique to determine mRNA levels in biological samples because of its sensitivity and large dynamic range. However, several issues like operator variability, differences in RNA extraction yield and variations in the reverse transcription and PCR reactions’ efficiencies must be considered to obtain reliable results [1,2,3]. In consequence, a normalization strategy is required to control for the experimental error that could be introduced during the whole procedure and could lead to biased results. Different strategies, like ensuring similar sample size prior to RNA extraction, submitting the same amount of RNA to reverse transcription or determining the expression levels of an internal control gene, are commonly used to reduce experimental error. Since there is no single procedure that controls for every error source, a combination is frequently adopted. The latter of these strategies is generally accepted to be the most suitable approach available since reference gene expression levels are measured in the same sample and subjected to almost the same sources of variability as the target genes under study [2].

Commonly named “housekeeping” genes were originally used as reference genes, since they had been selected as loading controls in Northern and Western blotting experiments for many years [2,3,4]. These genes encoding for proteins like actin, tubulin or GAPDH were assumed to be constitutively expressed and minimally regulated because they are required for basic cellular functions [4]. However, the use of more sensitive techniques like RT-qPCR demonstrated that the expression of these classic reference genes can vary extensively and that no single gene is able to fulfill the criteria required of a unique internal control [2,3,4,5,6,7,8,9]. Nevertheless, “housekeeping” genes, or any gene, could be used as an internal control as long as they have constant expression under the conditions described for the experiment in question [2]. If the chosen reference gene exhibits systematic differences in expression across sample subgroups, the results obtained would be completely biased. Furthermore, if it had a large expression error, the noise of the assay would be increased and the detection of small differences would become unachievable [10]. However, a limited extent of variation would be acceptable, depending on the degree of resolution required for the experiment [11]. In consequence, reference genes must be properly validated before performing any RT-qPCR experiment.

In order to measure expression levels accurately, normalization by a factor comprising multiple reference genes instead of one is suggested [6]. However, the number of genes to be used should be balanced between practical considerations and minimizing the variation in this factor [6,11,12]. Different algorithms were developed to analyze qPCR data to find the most stable genes among a group of candidates. The most frequently applied are geNorm [6], NormFinder [11], BestKeeper [12] and the Comparative Δ*C*q method [13].

geNorm analysis assumes that the expression ratio of two ideal reference genes must remain constant between samples, meaning that this ratio would not be affected by experimental conditions. It calculates a gene expression stability value, *M*, as the average pairwise variation of a particular gene with any other candidate. Genes with the lowest *M* values are the most stably expressed according to geNorm. Stepwise exclusion of the genes with the highest *M* value is performed until the most stable pair of genes is reached. Once reference genes are ranked according to their expression stability, the optimal number of genes required is determined and a normalization factor is calculated as the geometric mean of the expression levels of those genes [6]. The Comparative Δ*C*q method is a simpler version of geNorm. Based on the same principle, it calculates, for every sample, the difference between the quantification cycle values (Δ*C*q) of each possible combination of genes, which should remain constant among samples if both genes are stably expressed. By calculating the standard deviation (*SD*) of the Δ*C*q values between two genes obtained for all samples, and, then, the average standard deviations of the Δ*C*qs of each gene compared to all other genes, genes can be ranked according to expression stability [13]. In contrast, NormFinder is a model-based approach that analyzes not only the overall expression deviation, but also if the candidate reference gene shows systematic differences across sample subgroups. It estimates the intragroup and intergroup variation and combines them in a stability value, which represents a practical measure of the systematic error that would be introduced when using the investigated gene [11]. Finally, BestKeeper first performs an estimation of gene expression stability considering the *SD* of the quantification cycle (*C*q) values. Any gene with a *SD* smaller than 1 is used to calculate the “BestKeeper index” for each sample, which is the geometric mean of those *C*q values and should be used as a normalization factor. Furthermore, BestKeeper evaluates if potential reference genes correlate well with each other and only those highly correlated genes are recommended to be included in the BestKeeper index [12].

Growth hormone (GH) has a central role in the promotion of postnatal body growth and it is also involved in a variety of metabolic functions. It promotes longitudinal growth soon after the second week of life in rodents. In contrast, the first phase of rapid growth, which occurs immediately after birth, is GH-independent [14,15,16]. Consequently, transgenic mice overexpressing GH exhibit accelerated growth from the third week of age, in spite of having high circulating hormone levels since birth [17,18]. Previous experiments performed in our laboratory in liver of GH-transgenic mice revealed an age-dependent activation of GH signaling pathways leading to differences in the expression of many genes, like those encoding for insulin-like growth factor 1, negative regulators of GH signaling, cell cycle mediators and transcription factors [18,19]. Considering these results in liver, we aimed to determine the effects of GH in skeletal muscle to further address the molecular mechanisms engaged in the onset of GH action on somatic growth. Prior to evaluating the expression levels of genes related to GH signaling, validation of a proper normalization strategy was necessary.

The current work presents an exhaustive study of the reliability of potential reference genes to be used in the study of GH signaling in skeletal muscle of normal and GH-transgenic mice during their rapid growth period. The stability of a group of potential reference genes was determined by the algorithms previously mentioned and through the analysis of their relative expression levels. We demonstrate that, under certain experimental situations, these approaches could be not enough to support the election of suitable reference genes and thus the necessity of exploring other factors, not generally considered, emerges.

## 2. Results

### 2.1. Primer Specificity and Amplification Efficiency

Initial RT-qPCR quality control was assessed by product analysis. A single peak detected in the melting curve examination and a single band of expected size observed on the agarose gel confirmed the amplification of unique products by each primer pair and no primer dimer formation (Figures S1 and S2). Moreover, no amplification product was detected in the no-reverse transcription and no-template controls included in each reaction plate.

Amplification efficiencies were calculated from the slopes of the standard curves obtained by linear regression. These curves were constructed based on *C*q values from six serial cDNA dilutions of reversed transcribed RNA. Primer pairs showed an efficiency ranging from 91% to 108% with correlation coefficients higher than 0.98 (Table 1). Considering these results, an amount of cDNA that yielded an appropriate *C*q value for each gene was selected as initial template amount for qPCR experiments.

### 2.2. Selection of Potential Reference Genes for Studies in Skeletal Muscle of Normal and GH-Overexpressing Growing Mice

The first approach for candidate gene selection was based on our previous studies in the liver of GH-overexpressing transgenic and normal mice from the same line. Experiments performed in adult female mice used β-2 microglobulin (*B2M*) as a reference gene [20], whereas peptidylprolyl isomerase A (*PPIA*), also known as cyclophilin A, was used in studies in male and female growing and adult mice liver [18,19,21]. Consequently, both genes were included as potential reference genes for the current study in skeletal muscle of growing mice.

In order to ensure a convenient number of genes for proper validation, other genes were incorporated into the analysis: β-actin (*ACTB*), glyceraldehyde-3-phosphate dehydrogenase (*GAPDH*), hypoxanthine guanine phosphoribosyl transferase (*HPRT1*), 18S ribosomal RNA (*RN18S*), ribosomal protein L13A (*RPL13A*) and tyrosine 3-monooxygenase/tryptophan 5-monooxygenase activation protein, and zeta polypeptide (*YWHAZ*). This group comprises classic reference genes and also genes selected as the most stably expressed in published validation processes performed in heterogeneous sets of samples, including those derived from different tissues [6] or animals of different age [22,23,24]. Moreover, glycogen synthase kinase 3β (*GSK3B*) was included as well, since its protein content showed minimal variation in skeletal muscle of 2-, 4- and 9-week-old normal and transgenic mice in comparison to other GH-signaling related proteins previously evaluated (Figure S3). Special attention was paid to choosing genes involved in different biological processes in an attempt to avoid selection of co-regulated genes.

### 2.3. Evaluation of Potential Reference Gene Expression Stability in Skeletal Muscle of Normal and GH-Overexpressing Growing Mice

#### 2.3.1. Analysis of Quantification Cycle (*C*q) Values Dispersion and Determination of Expression Stability by Algorithms

**Figure 1:**
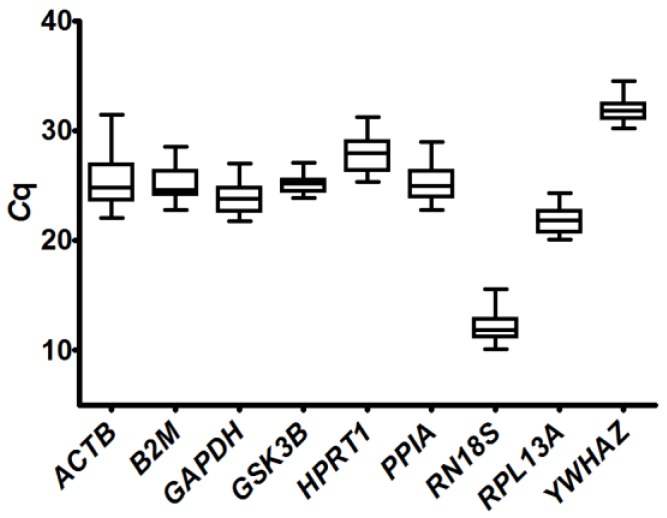
Quantification cycle (*C*q) values dispersion for each potential reference gene in skeletal muscle of growing mice. The expression levels of nine potential reference genes determined by reverse transcription-quantitative PCR (RT-qPCR) for the same amount of total reverse transcribed RNA are shown as medians (lines), 25th percentile to the 75th percentile (boxes) and ranges (whiskers) for 51 skeletal muscle samples of 2-, 4- and 9-week-old (2, 4, 9 w) normal (N) and transgenic (T) mice (2 wN: *n* = 9, 2 wT: *n* = 8, 4 wN: *n* = 10, 4 wT: *n* = 10, 9 wN: *n* = 6, 9 wT: *n* = 8).

An overview of the dispersion of raw *C*q values of nine potential reference genes is shown in Figure 1. Despite raw *C*q values needing to be transformed into relative quantities to acquire biological meaning, this kind of plots are informative as genes with less intragroup and intergroup variation will show less *C*q dispersion. Taking this into account, *GSK3B*, *RPL13A* and *YWHAZ*, which exhibited the least variation, seemed to be the most stable genes, while *ACTB* would be the most unstable with a difference between maximum and minimum *C*q of 9.

Although this overall analysis would reject the use of some of the evaluated genes to calculate a normalization factor, we determined the expression stability of the whole group of genes. Different available algorithms were used to address this issue: BestKeeper, NormFinder, geNorm and the Comparative Δ*C*q method. BestKeeper and the Comparative Δ*C*q method require data expressed as raw *C*q values while NormFinder and geNorm analyses need *C*q values to be transformed into relative expression data. The stability values obtained for each gene are shown in Table 2.

According to BestKeeper, the most stable gene was *GSK3B*, followed by *YWHAZ* and *RPL13A*. To calculate the BestKeeper index, which is used as a normalization factor, *RN18S* should also be included since it displayed an *SD* smaller than 1. In contrast, *GAPDH*, *B2M*, *PPIA*, *HPRT1* and *ACTB* were not stable enough to be considered. geNorm and the Comparative Δ*C*q method results were in good agreement with BestKeeper ranking, since they also found that the most stable genes were *RPL13A*, *YWHAZ*, *GSK3B* and *RN18S*. Seven genes among the nine tested exhibited an average expression stability value (*M*) lower than 1, which is recommended for heterogeneous sample sets by geNorm developers [25]. On the other hand, NormFinder ranking exhibited a few discrepancies as *PPIA* was included among the four first places and *GSK3B* was placed in fifth position. However, it also ranked *RPL13A*, *YWHAZ* and *RN18S* between the first four places regarding expression stability like the other algorithms. In summary, three out of four algorithms agreed in ranking *RPL13A*, *YWHAZ*, *GSK3B* and *RN18S* as the most stable genes and all of them coincided in *ACTB* being the most unstable. Furthermore, these results were in good agreement with those obtained from the overall analysis of the *C*q values’ dispersion.

In order to determine the most stable genes regarding only the age effect, results from normal mice were considered separately, and were subjected to an independent analysis by the same algorithms (Table 3). In this case, geNorm, BestKeeper and the Comparative Δ*C*q method also determined *RPL13A*, *YWHAZ*, *GSK3B* and *RN18S* as the most stably expressed genes. Similar to the analysis of data from both genotypes, NormFinder led to a rather different ranking with *GAPDH* placed in a better position regarding to expression stability.

#### 2.3.2. Analysis of Relative Expression Levels

**Figure 2:**
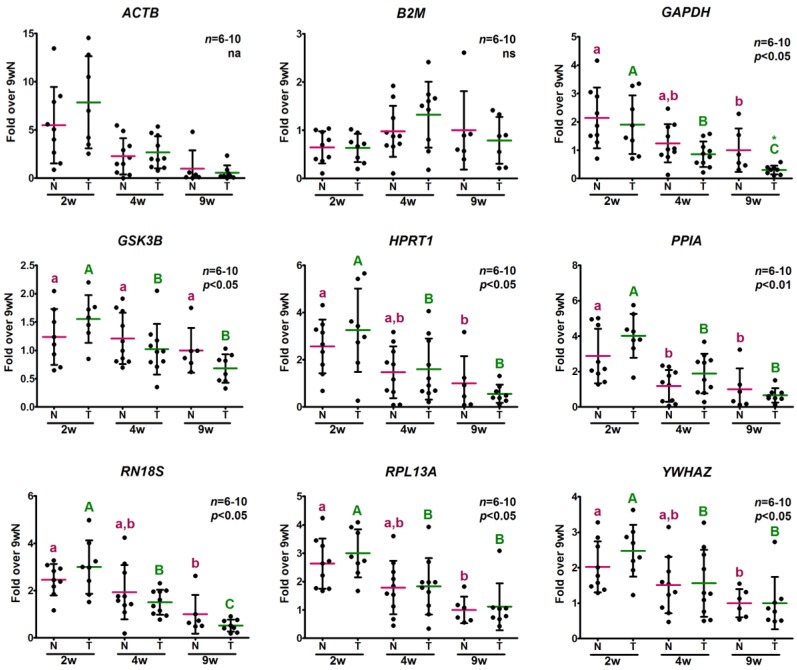
Relative expression levels of potential reference genes determined for the same amount of total RNA. The relative expression levels of nine potential reference genes were determined by RT-qPCR in samples obtained from the same amount of reverse transcribed total RNA from skeletal muscle of 2-, 4- and 9-week-old (2, 4, 9 w) normal (N) and transgenic (T) mice. Data are the mean ± *SD* (standard deviation) of the indicated *n* number of samples per group, each one representing a different animal. Normal distribution was evaluated by the D’Agostino-Pearson test; *B2M* and *GAPDH* data were log-transformed to achieve normality. *ACTB* log-transformed data did not pass normality test and were not further analyzed (na). Normally distributed data were analyzed by two-way ANOVA followed by the Bonferroni post-test. Different letters denote significant differences by age; small letters correspond to normal mice and capital letters to transgenic animals. Asterisks indicate differences between genotypes. A *p*-value lower than 0.05 was considered statistically significant. na stands for not analyzed. ns stands for non-significant.

In a different approach to analyze the stability of the candidate reference genes, the distribution of the relative expression levels was valuated. To be used as internal controls, gene expression must be independent of the experimental conditions and, consequently, no significant differences should be expected between sample subgroups. Interestingly, the whole group of genes analyzed, except for *B2M*, exhibited significant differences between ages, with highest expression in 2-week-old mice followed by a decline (Figure 2). However, each gene displayed a different degree of change with age. *ACTB* yielded the greatest difference with an approximately seven-fold decrease in 9-week-old mice in comparison to weanlings. On the other hand, *GSK3B* exhibited the smallest age variation. With regard to *B2M*, despite the relative expression level differences between experimental groups not achieving statistical significance, the higher expression observed in 4-week-old transgenic samples would bias results if any target gene expression was to be normalized by *B2M* levels, especially if small differences were expected. Therefore, none of the genes evaluated would be reliable to be used as an internal control according to this approach. If only normal mice were to be analyzed, *B2M*, as well as *GSK3B*, would be suitable to calculate the normalization factor, as they did not exhibit significant differences between ages.

### 2.4. Determination of Potential Reference Gene Expression Levels in Isolated Skeletal Muscle mRNA from Normal Mice

**Figure 3:**
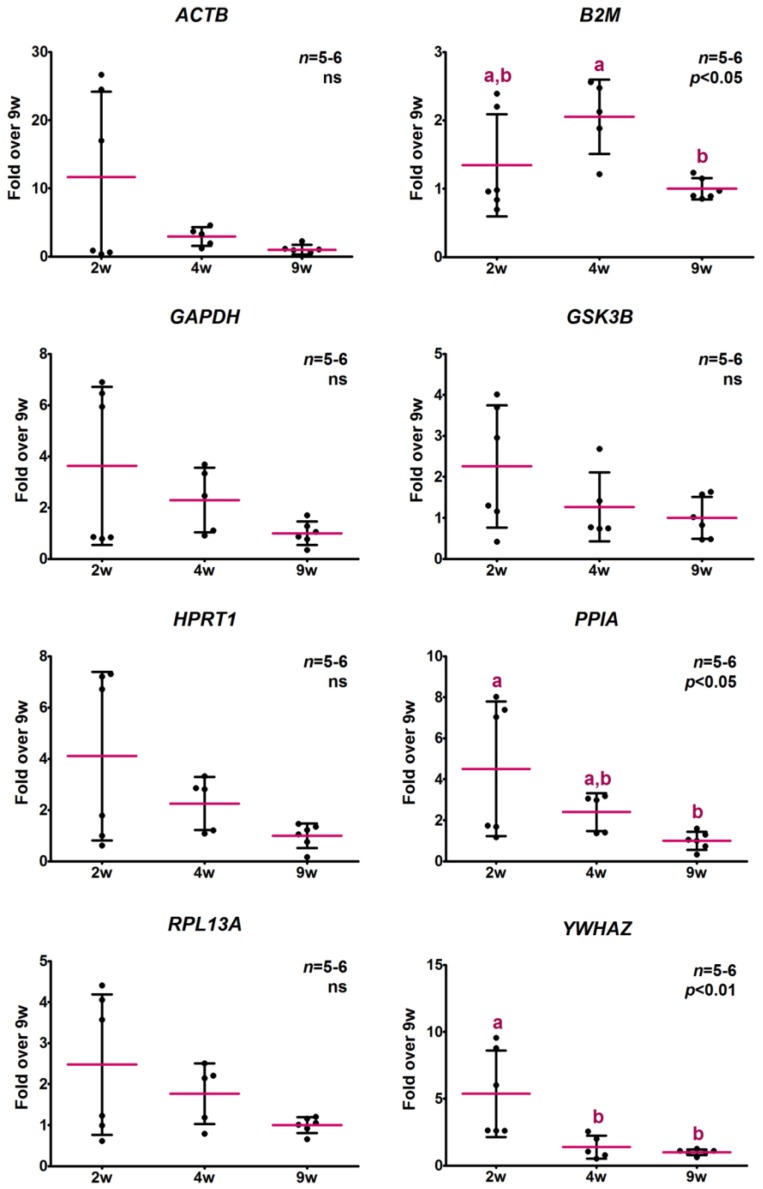
Relative expression levels of potential reference genes determined for the same amount of isolated mRNA. The relative expression levels of eight potential reference genes were determined by RT-qPCR in samples obtained from the same amount of reverse transcribed isolated messenger RNA from skeletal muscle of 2-, 4- and 9-week-old (2, 4, 9 w) normal mice. Data are the mean ± *SD* of the indicated *n* number of samples per group, each one representing a different animal. Normal distribution was evaluated by the D’Agostino-Pearson test and *ACTB*, *HPRT1*, *PPIA* and *YWHAZ* data were log-transformed to achieve normality. Different letters denote significant differences by age determined by one-way ANOVA followed by the Bonferroni post-test. A *p*-value lower than 0.05 was considered statistically significant. ns stands for non-significant.

Considering that almost every gene displayed higher expression levels in 2-week-old mice, we wondered if this could be due to alterations in the mRNA to total RNA ratio among samples from mice of different ages. Since gene expression levels were measured in cDNA samples obtained from the same amount of total RNA, an alteration in the proportion of mRNA could be biasing results. To address this issue, RNA extraction and subsequent mRNA isolation from skeletal muscle of 2-, 4- and 9-week-old normal mice was performed. The expression levels of the candidate genes under study were measured by qPCR in cDNA samples obtained from the same amount of isolated mRNA. The ribosomal RNA *RN18S* was also determined as a control of the isolation procedure and negligible levels were obtained. As it is shown in Figure 3, every potential reference gene exhibited relative expression profiles similar to those previously obtained with total reverse transcribed RNA. However, for the majority of the measured genes, the apparent age-related differences were not statistically significant as a consequence of the higher dispersion of the results.

### 2.5. Analysis of the Relative Expression Levels of Target Genes Normalized against the Selected Reference Genes

**Figure 4:**
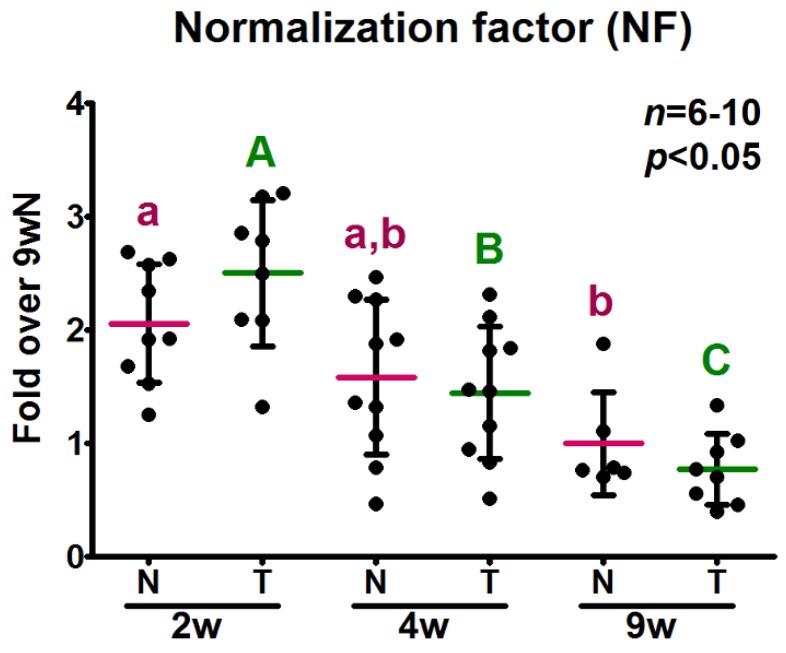
Normalization factor calculated as the geometric mean of the most stable genes according to algorithms. The geometric mean of the relative expression levels of *GSK3B*, *YWHAZ*, *RPL13A* and *RN18S*, determined by RT-qPCR in samples obtained from the same amount of reverse transcribed total RNA from skeletal muscle of 2-, 4- and 9-week-old (2, 4, 9 w) normal (N) and transgenic (T) mice, was calculated for each sample to obtain the corresponding normalization factor. Data are the mean ± *SD* of the indicated *n* number of samples per group, each one representing a different animal. Normal distribution was confirmed by the D’Agostino-Pearson test before analysis by two-way ANOVA followed by the Bonferroni post-test. Different letters denote significant differences by age; small letters correspond to normal mice and capital letters to transgenic animals. A *p*-value lower than 0.05 was considered statistically significant.

In order to evaluate the effect of normalization by the most stable genes according to algorithms, we determined the relative expression levels of two genes of interest in the study of GH signaling in skeletal muscle. Consequently, the growth hormone receptor (*GHR*) and insulin-like growth factor 1 (*IGF1*) mRNA levels were measured. The normalization factor (NF) for each sample was calculated as the geometric mean of the relative expression levels of the top four genes according to three out of four algorithms: *RPL13A*, *YWHAZ*, *GSK3B* and *RN18S*, since, in most cases, three to five genes are required for accurate normalization [26]. As it could have been predicted in view of the relative expression levels of the reference genes considered, the NF mean decreased with age (Figure 4).

**Figure 5:**
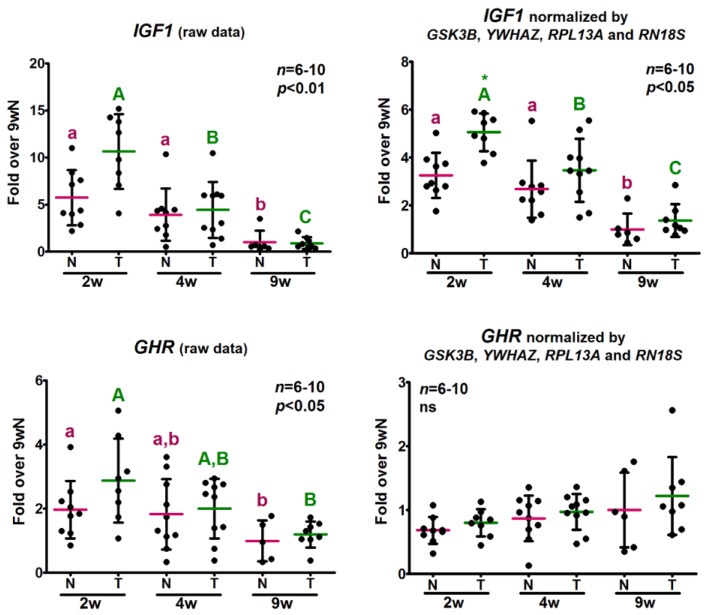
Effect of correction by the normalization factor in the relative expression levels of target genes. The relative expression levels of GH-signaling related genes were determined by RT-qPCR in samples obtained from the same amount of reverse transcribed total RNA from skeletal muscle of 2-, 4- and 9-week-old (2, 4, 9 w) normal (N) and transgenic (T) mice. *IGF1* and *GHR* relative expression levels are shown as raw data and normalized by *GSK3B*, *YWHAZ*, *RPL13A* and *RN18S* expression levels. Data are the mean ± *SD* of the indicated *n* number of samples per group, each one representing a different animal. Normal distribution was evaluated by the D’Agostino-Pearson test and all data, except that for *GHR* corrected by NF, were log-transformed to achieve normality. Normally distributed data were analyzed by two-way ANOVA followed by the Bonferroni post-test. Different letters denote significant differences by age; small letters correspond to normal mice and capital letters to transgenic animals. Asterisks indicate differences between genotypes. A *p*-value lower than 0.05 was considered statistically significant. ns stands for non-significant.

The gene expression profiles of *GHR* and *IGF1* are presented in Figure 5, both as raw data and as normalized by *GSK3B*, *YWHAZ*, *RPL13A* and *RN18S*. *IGF1* expression levels showed higher mRNA amounts in young mice compared to adults, either as raw or normalized data. Significant differences were found between genotypes in 2-week-old animals only after normalization. Despite the correction with NF attenuated the change in mRNA levels with age, results were similar in terms of differences among groups. On the other hand, while non-normalized *GHR* levels exhibited a decreasing trend with age normalization by NF changed this profile and an increasing tendency in *GHR* mRNA amounts was observed, although it did not achieve statistical significance. Noticeably, the trend with age displayed by the non-normalized relative expression levels of both target genes was similar to the results obtained for almost every potential reference gene as their expression declined with age.

## 3. Discussion

Quantitative PCR is one of the most powerful techniques used to detect and determine the amount of nucleic acids in different kinds of samples. Therefore, it is widely applied in life sciences, molecular diagnostics, agriculture and medicine [27,28]. Quantitative PCR preceded by reverse transcription (RT-qPCR) is commonly used in the evaluation of gene expression patterns. However, in order to obtain reliable and biologically meaningful RT-qPCR results, it is necessary to minimize the effect of any non-biological variation. This is commonly accomplished by the assessment of reference gene expression levels, which is subjected to almost the same experimental error as quantification of target gene mRNA levels [7,28]. The expression of these internal controls must not be affected by the experimental conditions under study [2,7,28]. Despite normalization against reference genes being the most widely accepted strategy to control for experimental error, it must be considered that, unless reference and target genes are measured together in a multiplex reaction, differences in the amount of input cDNA would not be taken into account. Moreover, when mRNA levels are reported relative to those of reference genes, both genes must be amplified with similar efficiencies for the determinations to be compared [28].

There is a general agreement in that the use of more than one reference gene leads to more reliable results [4,6,28], and it also allows an evaluation of the stability of these transcripts [25]. The group of potential reference genes assessed in this work comprises classical “housekeeping” genes (*ACTB*, *GAPDH*, *RN18S*), genes which were found to be stably expressed in heterogeneous groups of samples (*HPRT1*, *RPL13A*, *YWHAZ*), genes already used as control in the same animal model but in a different tissue (*B2M*, *PPIA*) and genes encoding for proteins that exhibit minimal differences in their expression levels in the samples under study (*GSK3B*). Therefore, the list of candidates includes genes belonging to different functional classes and a proper number to analyze expression stability [11].

In the current study, a thorough analysis was carried out to determine which genes are the most stably expressed and, hence, should be used for normalization of relative expression levels of genes expressed in skeletal muscle of normal and GH-overexpressing mice at different ages. First, an overall evaluation of *C*q values was conducted and RT-qPCR data were processed with geNorm, NormFinder, BestKeeper and the Comparative Δ*C*q method algorithms. BestKeeper and geNorm mostly agreed in their ranking, with the exception of *GSK3B* and *B2M*, which were ranked higher by BestKeeper than by geNorm. The Comparative Δ*C*q method led to a similar ranking as geNorm, which was expected considering that they are both based on the comparison of the differences between every possible combination of genes for each sample. The most noteworthy discrepancy was that the Comparative Δ*C*q method found *GAPDH* as one of the least stably expressed genes, while geNorm ranked it in the fourth position among the nine tested genes. However, since geNorm analyzes qPCR data expressed as fold change relative to a calibrator sample and the Comparative Δ*C*q method evaluates raw *C*q values, only genes with similar *C*q values could be analyzed with high accuracy by the Comparative Δ*C*q method [13]. In the current study, there were large differences in the *C*q values obtained for the evaluated genes (Figure 1), which could be the reason for the discrepancies found between these two algorithms. When qPCR data were analyzed with NormFinder, a slightly different ranking was obtained. In comparison to the results of the other algorithms, *RN18S* and *PPIA* were placed in a better position and *GSK3B* was ranked lowest according to expression stability. Since NormFinder stability value considers not only the intragroup but also the intergroup variation, unlike the rest of the algorithms that do not distinguish between experimental groups, some differences could be expected.

Furthermore, a statistical analysis of the relative expression levels of potential reference genes was performed and compared with the results of the algorithms. The majority of the genes evaluated exhibited significant age differences in their relative expression levels, except for *B2M* (Figure 2). Considering that the expression of an internal control gene must remain constant between experimental conditions, these differences among ages in the relative expression levels of the genes evaluated would reject their use. Despite the differences between experimental conditions in *B2M* relative expression levels did not achieve statistical significance, its use as a correction factor will also bias the result of any target gene normalized against it, especially in transgenic mice. *ACTB* was not included for this statistical analysis, since it was already determined as the most unstable gene by the overall evaluation of *C*q values and algorithms and it would have required a non-parametric analysis since it did not pass the normality test performed, although it is evident that its relative expression levels were deeply affected by age.

*B2M* exhibited an intergroup variation similar to that of *GSK3B* as they both displayed an approximately twofold change between the mean of the experimental conditions with the highest and the lowest expression levels, while the other genes showed a greater change. However, while *GSK3B* was ranked among the first places by three out of four algorithms, *B2M* always emerged as one of the least stably expressed genes. This discrepancy could be explained, at least in part, when their expression level profiles were compared to those of the other genes evaluated. While *GSK3B* displayed a decreasing trend with age like the other genes, *B2M* exhibited a completely different profile. Considering the stability value definition of geNorm and the Comparative Δ*C*q method, the outcome of these algorithms is easily affected by genes that behave all in the same way. These limitations have also been reported by others [29,30] and a further discussion about this topic is provided as a Supplementary File (File S1).

While intragroup variation could be improved by adequate biological replication and a careful control of input cDNA levels, intergroup variation must always be considered as it could invalidate the use of any gene as an internal control. Therefore, it is necessary to determine if the experimental conditions under study affect the expression of potential reference genes. Despite the fact that the stability values obtained are below the recommended cut-off or resemble the commonly reported values for the most stable reference gene in published data sets, it cannot be ruled out that there is systematic variation among sample subgroups even when using algorithms that consider the intergroup variation. After a cautious selection of a group of candidate genes, an overall analysis of the relative expression levels, like the one performed in this study, could be enough to estimate the intergroup variation.

In normal and transgenic mouse skeletal muscle, the relative expression levels of every gene under study decreased with age, with the exception of *B2M* (Figure 2). A systematic time course change in mRNA levels of genes evaluated as potential reference genes was also found in previous studies [31,32,33,34,35]. There is vast evidence supporting that development of organisms, differentiation and cell growth induce changes in the number of transcripts of many genes. However, the authors of those studies suggested that the impact on the expression of classic reference genes was indeed an artifact of the RT-qPCR protocol, considering that it is usually performed with the same amount of total reverse transcribed RNA of each sample and, if there is a large and significant change in the level of any RNA species, the relative mRNA abundance of other transcripts may appear concentrated or diluted. Therefore, the decline in gene expression observed over time could be the result of a dramatic increase of something else composing total RNA that would be “diluting” the measured mRNA (“the dilution effect”). These authors arrived at these conclusions since they observed that, when every potential reference gene decreased, the total amount of RNA extracted presented a peak and/or there was a large increment in the expression of tissue specific genes that could generate the dilution of the others [31,32,34].

To normalize for this “dilution effect”, these authors selected those genes with the best stability values and used them to calculate a normalization factor, despite a time effect on their relative expression levels. This strategy is correct only if the copy number per cell of those reference genes is not affected by experimental conditions and the variation observed in the relative expression levels is only due to the change of other mRNA species in the opposite direction or a variation in something else composing total RNA that significantly affects the amount of total RNA per cell and causes the “dilution”. However, when the same amount of total RNA is reverse transcribed, the “dilution effect” would cause a distortion in relative expression levels that could not be distinguished from the situation in which there is only a specific change in the transcription of that potential reference gene. Consequently, it is mandatory to further analyze this situation prior to determining the normalization strategy to apply.

Previous evidence indicates that the amount of ribosomal RNA could be altered in tumors [36,37] or affected by heat shock treatment in bacteria [38] or gene therapy in mice [39]. Moreover, it was also described that there could be an overall increase or decrease in transcription, which means that the total amount of mRNA could vary with experimental conditions [40]. In order to exclude the possibility that a variation in the proportion of each RNA type could distort results, experiments could be performed with cDNA obtained from the same amount of mRNA, instead of total RNA. Taking this into account and considering the results of the current study, we wondered if there was a change during growth in the ratio between mRNA and total RNA that could explain the consistent decrease in expression levels of almost every reference gene. To evaluate this, we measured the expression levels of the group of potential reference genes in cDNA samples obtained from the same amount of previously isolated mRNA.

Similar results were obtained when RT-qPCR was performed with purified mRNA in comparison to those obtained for total RNA (Figure 3). If the higher gene expression previously observed in skeletal muscle of 2-week-old mice was only a consequence of a larger proportion of mRNA at that age, no differences would be expected among ages in the measured levels of those genes when RT-qPCR was performed with isolated mRNA. However, all potential reference genes exhibited similar profiles to those previously obtained for total RNA, despite statistical significance not always being achieved, probably because of the smaller number of samples tested for each condition and the higher global error introduced by a more complex procedure.

These results suggest that a variation in the proportion of mRNA in total RNA is not responsible for the disparity found in the expression levels of almost every gene with age. Therefore, the consistent change observed could not be a consequence of a dilution effect caused by an increase of any RNA species different from mRNA or a consequence of a decrease in overall transcription. However, there is still another possible situation that could not be dismissed in the interpretation of these results. If there was a large increment with age in the expression of any gene that significantly enlarged the amount of total RNA per gram of tissue, a consistent decrease in the relative expression levels of every other gene would be observed even when determined in the same amount of reverse transcribed mRNA. In this situation, the relative expression levels of reference genes would be affected in the same way and therefore would be useful for correcting results for this dilution effect.

In summary, if any dilution effect is occurring, as a consequence of a significant increase in any mRNA or other RNA species that affects the total amount of RNA, only reference genes that reflect this effect are suitable to be used as internal controls, as it was previously reported [31,32,33,34]. Furthermore, the use of reference genes for this purpose would also be adequate if there was an overall change in mRNA transcription between samples that affected every gene to the same extent. In that case, the relative expression levels of true reference genes would only be affected by this change in transcriptional activity and, consequently, these levels would correlate with the total amount of mRNA present in each sample. Therefore, the use of reference genes to calculate a normalization factor would correct for alterations in transcriptional activity and it would allow the comparison of target gene expression levels in samples with different transcriptional activities. This kind of study could also be performed if equal amounts of reverse transcribed mRNA were used for qPCR experiments. However, it must be taken into account that, if the aim of the study was to detect changes in general transcriptional activity between experimental conditions, this could only be achieved if results were obtained for total RNA and not normalized with reference genes. Finally, it must always be considered that none of these situations could be distinguished from that where the experimental conditions affect specifically the transcription of potential reference genes. However, an exhaustive analysis of many unrelated potential reference genes would permit determining whether any of the effects previously mentioned is occurring, i.e., a dilution effect or alterations in general transcriptional activity, and it would support the selection of the best normalization strategy.

Considering the results of the current study, we could reject the hypothesis of a systematic change with age in almost every potential reference gene occurring due to alterations in the overall cell transcriptional activity or in the amount of something composing total RNA that alters mRNA proportion in the samples. Moreover, it could be expected that eight unrelated genes would rarely be all affected in the same way by the experimental conditions under study. Consequently, we suggest that the observed changes in the relative expression levels of those genes could reflect a large increment in any mRNA that is not being measured in this work.

Since any target gene would be affected in the same way, as it could be observed for raw data from *GHR* and *IGF1* that exhibited the same age-dependent profile as almost every potential reference gene, its relative expression levels should be normalized by these variable reference genes that reflect the dilution effect. The effect of this normalization will depend on the degree of change in target gene expression levels among experimental conditions. If this change was considerable, as it occurred with *IGF1*, the dilution effect would not significantly affect it, and a similar outcome would be obtained after normalization. On the contrary, if target gene expression levels exhibited minor variation with experimental conditions, completely different results could be obtained depending on the normalization factor chosen. For instance, when *GHR* results were corrected, the decreasing variation that it displayed prior to normalization disappeared and no significant differences were detected among ages and genotypes. The degree of change observed before normalization is similar to that of the calculated normalization factor, which means that the relative expression levels of *GHR* were mainly altered because of the dilution effect.

## 4. Materials and Methods

### 4.1. Animals

Transgenic mice used in this study were derived from animals kindly provided by Thomas E. Wagner and Jun S. Yun (Ohio University, Athens, OH, USA) and were expressing bovine *GH* gene linked to regulatory regions of the rat phosphoenolpyruvate carboxykinase (*PEPCK*) gene. The breeding system followed in order to obtain transgenic mice, as well as animal housing and feeding conditions, were previously described [18,19]. Mice were killed by cervical dislocation under isofluorane anesthesia, and skeletal muscle from the hind limbs was removed and stored at −70 °C until use. The appropriateness of the experimental procedure, the required number of animals used and the method of acquisition were in compliance with federal and local laws. The protocol was approved by the Laboratory Animal Care and Use Committee (LACUC) of Southern Illinois University (Springfield, IL, USA) (Permit Number: 178-02-001. Original approval date: 08/23/2002. Date of continuing review approval: 08/23/2014).

### 4.2. Experimental Design

Male and female mice of three representative ages were used: 2-week-old, just before the GH-induced growth spurt, when both genotypes cannot be distinguished by their body size; 4-week-old, during the GH-dependent rapid phase of growth, when transgenic mice display greater body size than normal mice; and 9-week-old, the young adult mice used as reference [18]. Non-transgenic siblings were used as the control group. Consequently, there were six experimental groups: two, four and nine week-old normal and transgenic mice, each subset of samples composed of approximately half males and half females reaching a total of 6 to 10 samples per group.

### 4.3. RNA Extraction and Reverse Transcription (RT)

Total RNA was extracted from 50 mg of tissue using TRIzol Reagent (Invitrogen^TM^, Waltham, MA, USA) following the manufacturer’s instructions. The product was quantified by measuring its absorbance at 260 nm and its purity was evaluated by its absorbance ratios at 260/280 nm and 260/230 nm using a NanoDrop 2000 UV-Vis Spectrophotometer (Thermo Fisher Scientific, Inc., Waltham, MA, USA). RNA integrity was assessed by 2% agarose gel. Samples with sub-optimal absorbance ratios or evidence of degradation on agarose gels were rejected from this study.

A total amount of 2 μg of RNA was treated with DNase I Amplification Grade (Invitrogen^TM^, Waltham, MA, USA) to eliminate remaining genomic DNA and further subjected to reverse transcription using iScript^TM^ cDNA Synthesis Kit (Bio-Rad Laboratories, Inc., Hercules, CA, USA) according to the manufacturer’s instructions. Additional samples were incubated under the same conditions without reverse transcriptase to validate DNase treatment.

### 4.4. Messenger RNA (mRNA) Isolation

High quality RNA (40 µg) from skeletal muscle of 2-, 4- and 9-week-old normal mice, obtained as previously described, was subjected to oligo(dT)-cellulose affinity chromatography in order to isolate polyadenylated (poly(A)^+^) mRNA (GenElute™ mRNA Miniprep Kit, Sigma-Aldrich, Inc., St. Louis, MO, USA). The product was quantified by measuring its absorbance at 260 nm. To obtain the corresponding cDNA, poly(A)^+^ mRNA (20 ng) was subjected to DNase treatment and reverse transcription as described for total RNA. No-reverse transcription controls were also performed.

Assays carried out with isolated mRNA were performed only in skeletal muscle samples from normal animals, leading to three groups consisting in 5 to 6 samples per group, approximately half males and half females.

### 4.5. Primer Design

Primers were designed with the available free software Primer BLAST [41] and OligoPerfect^TM^ Designer (Thermo Fisher Scientific, Inc.) following general recommendations: 55–65 °C melting temperature, 18–25 base pair length, 45–60% GC content and delimiting a 50–150 base pairs length amplicon. Primers that fulfilled these criteria were chosen, with the exception of *GAPDH* forward primer that contains 67% of GC and *ACTB* primer pair that amplifies a 319 base pairs length sequence (Table 4 and Table 5). To avoid amplification of potentially remaining genomic DNA, primers that span exon–exon junctions were chosen whenever possible. Moreover, no-reverse transcription controls were always performed and showed no amplification product.

Primer and amplicon secondary structures were evaluated using OligoAnalyzer 3.1 Software (Integrated DNA Technologies, Inc., Coralville, IA, USA) in order to select primers that do not form hairpins, self-dimers or heterodimers and that do not anneal to amplicon regions involved in hairpins. In addition, primers that amplified regions that could form stable secondary structures were rejected. Specificity of primers was checked by carrying out a BLAST search with Primer BLAST software. Primers were obtained from Integrated DNA Technologies, Inc. and Invitrogen^TM^.

### 4.6. Quantitative PCR (qPCR)

qPCR reaction was performed in an Applied Biosystems^®^ 7500 Real-Time PCR System with 300 nM of forward and reverse primers and 1× SYBR^®^ Select Master Mix (Applied Biosystems^TM^, Beverly, MA, USA) in a final reaction volume of 13 μL. Non-template and no-reverse transcription controls were included as recommended [1,28]. The following cycling conditions were used: 2 min at 50 °C and 2 min at 95 °C for enzyme activation, followed by 40 cycles of 15 s denaturing step at 95 °C and 1 min annealing and extension step at 60 °C. For proper *YWHAZ* amplification, an additional step of 30 s at 77 °C was added at the end of each cycle. After each run, dissociation curves were generated in order to detect non-specific products. Results were confirmed by running PCR products on a 2% agarose gel.

Standard curves were constructed from serial dilutions of a pool of cDNA samples of 2-, 4- and 9-week-old normal animals to evaluate the amplification efficiency (E% = (10^(−1/slope)^ − 1) × 100%) of each pair of primers. Six 2-fold serial dilutions starting from 8 ng/µL of total reverse transcribed RNA were prepared for this purpose, with the exception of *RN18S* curve that started with a 1.6 ng/µL dilution. Considering the results of these curves, a total amount of 2 to 20 ng of reverse transcribed total RNA or 0.3 to 0.8 ng of reverse transcribed poly(A)^+^ mRNA were used in each qPCR experiment to ensure an adequate *C*q value (lower than 35) for each gene. In these experiments, each biological replicate was analyzed in duplicate and, in order to evaluate as many samples as possible in the same plate, each gene was determined in independent qPCR runs [25]. For those experiments performed in total RNA, where 51 samples were under study, two qPCR experiments were run for each gene, adjusting the same *C*q threshold for both runs.

This method was also used for the transgene (*bGH*) detection, since 2-week-old transgenic mice cannot be differentiated by body size from their normal siblings. The procedure and the sequence of the primers employed have been previously described [18].

### 4.7. Data Analysis

Quantification cycle (*C*q) determination was performed with the Applied Biosystems^®^ 7500 Software v2.0.6 with automatic baseline correction and threshold setting. Prior to analyzing *C*q data, the automatic threshold setting was evaluated and, if needed, adjusted according to general recommendations. Relative gene expression levels were calculated by the comparative *C*q method [42], which refers results from experimental samples to a calibrator according to the equation E^A−B^ (E = amplification efficiency = 10^(−1/slope)^, A = average *C*q value of the corresponding run, B = mean *C*q value of the sample). Data were then expressed as fold change related to the mean of 9-week-old normal mice and analyzed by the D’Agostino-Pearson normality test to assess for Gaussian distribution. For those data sets that did not pass this test, logarithmic transformation was performed to achieve normality. The significance of the difference in the mean level for each transcript among ages and genotypes was assessed by two-way analysis of variance (ANOVA) or by one-way ANOVA when only normal mice were analyzed, followed by the Bonferroni post-test where a *p*-value lower than 0.05 was considered statistically significant. Differences among ages are expressed by different letters, small letters for normal animals and capital letters for transgenic mice. Differences between genotypes are expressed by an asterisk. This statistical analysis was performed using the GraphPad Prism^TM^ 5.01 program (GraphPad Software, Inc., La Jolla, CA, USA).

In order to determine the most stable genes among the set of potential reference genes, data were also analyzed with different statistical algorithms available as visual basic applications for Microsoft Excel: geNorm (version 3) [6], NormFinder (version 0953) [11], BestKeeper (version 1) [12] and the Comparative Δ*C*q method [13]. These methods yield a ranking based on stability values for each gene.

## 5. Conclusions

In summary, in RT-qPCR studies of gene expression, a careful selection of a group of candidate genes must be conducted prior to applying any validation strategy. The algorithms geNorm, NormFinder, BestKeeper and the Comparative Δ*C*q method are useful tools to determine which genes are the most suitable to be used as internal controls. However, stability values obtained with these programs are only meaningful in context and do not imply that there is no systematic change in mRNA amounts associated to experimental conditions. Therefore, it is mandatory to analyze relative expression levels to correct for this. Although only those genes whose transcription is not affected by experimental conditions must be used as reference genes, a consistent change in their relative expression levels does not invalidate their use. Before rejecting those variable genes from the study, the possibility of a change in overall transcription activity or a “dilution effect” altering the ratio between mRNA of reference genes and the total amount of RNA present in each sample must be considered. If this is the case, it is necessary to further analyze this situation before deciding which normalization strategy is the most appropriate to apply. This analysis requires the evaluation of a considerable number of genes to reach reliable conclusions. As was previously discussed, the use of reference genes is not always appropriate and, therefore, other normalization strategies should be considered.

On the basis of the present findings, we recommend the use of a factor comprising *GSK3B*, *YWHAZ*, *RPL13A* and *RN18S* for normalizing RT-qPCR data from skeletal muscle of growing mice, as these four genes reflect the “dilution effect” generated in this tissue during growth and were determined as the most stable among a group of nine genes by the algorithms applied.

## Figures and Tables

**Table 1:** Amplification efficiencies and *R*^2^ values of standard curves of potential reference genes and target genes for reverse transcription-quantitative PCR (RT-qPCR) experiments in skeletal muscle of growing mice. Standard curves were constructed from six cDNA dilutions of a pool of samples of 2-, 4- and 9-week-old normal animals. Amplification efficiencies of each pair of primers were calculated from the slope of the curves, obtained by linear regression, according to the equation E% = (10^(−1/slope)^ − 1) × 100%.

Gene Symbol	Efficiency (%)	*R*^2^
*ACTB*	101	0.982
*B2M*	98	0.998
*GAPDH*	95	0.999
*GHR*	93	0.995
*GSK3B*	91	0.995
*HPRT1*	105	0.991
*IGF1*	108	0.996
*PPIA*	101	0.998
*RN18S*	101	0.998
*RPL13A*	100	0.998
*YWHAZ*	99	0.980

**Table 2:** Expression stability values of potential reference genes for RT-qPCR experiments in skeletal muscle of normal and growth hormone (GH)-overexpressing growing mice calculated by available algorithms. Stability values were defined by the developers of each algorithm and were calculated with the available software programs geNorm, BestKeeper, the Comparative Δ*C*q method and NormFinder. Rankings are established in descending order of expression stability. A total of 51 skeletal muscle samples of 2-, 4- and 9-week-old (2, 4, 9 w) normal (N) and transgenic (T) mice were subjected to this analysis (2 wN: *n* = 9, 2 wT: *n* = 8, 4 wN: *n* = 10, 4 wT: *n* = 10, 9 wN: *n* = 6, 9 wT: *n* = 8).

Ranking	Bestkeeper		geNorm		Comparative Δ*C*q		NormFinder	
	Gene Symbol	Stability Value	Gene Symbol	Stability Value	Gene Symbol	Stability Value	Gene Symbol	Stability Value
1	*GSK3B*	0.587	*RPL13A*/*YWHAZ*	0.458	*RPL13A*	1.073	*RPL13A*	0.202
2	*YWHAZ*	0.762	*GSK3B*	0.549	*YWHAZ*	1.092	*RN18S*	0.279
3	*RPL13A*	0.916	*RN18S*	0.698	*GSK3B*	1.146	*YWHAZ*	0.291
4	*RN18S*	0.962	*GAPDH*	0.757	*RN18S*	1.159	*PPIA*	0.398
5	*GAPDH*	1.194	*PPIA*	0.871	*PPIA*	1.279	*GSK3B*	0.409
6	*B2M*	1.263	*HPRTA*	0.989	*HPRT1*	1.427	*HPRT1*	0.420
7	*PPIA*	1.317	*B2M*	1.104	*GAPDH*	1.479	*GAPDH*	0.421
8	*HPRT1*	1.399	*ACTB*	1.279	*B2M*	1.780	*B2M*	0.762
9	*ACTB*	1.958	-	-	*ACTB*	1.891	*ACTB*	0.822

**Table 3:** Expression stability values of potential reference genes for RT-qPCR experiments in skeletal muscle of normal growing mice calculated by available algorithms. Stability values were defined by the developers of each algorithm and were calculated with the available software programs geNorm, BestKeeper, the Comparative Δ*C*q method and NormFinder. Rankings are established in descending order of expression stability. A total of 25 skeletal muscle samples of 2-week-old (*n* = 9), 4-week-old (*n* = 10) and 9-week-old (*n* = 6) normal mice were subjected to this analysis.

Ranking	Bestkeeper		geNorm		Comparative Δ*C*q		NormFinder	
	Gene Symbol	Stability Value	Gene Symbol	Stability Value	Gene Symbol	Stability Value	Gene Symbol	Stability Value
1	*GSK3B*	0.646	*RPL13A*/*YWHAZ*	0.518	*YWHAZ*	1.173	*RPL13A*	0.169
2	*YWHAZ*	0.796	*GSK3B*	0.593	*RPL13A*	1.188	*RN18S*	0.227
3	*RN18S*	0.952	*RN18S*	0.701	*RN18S*	1.197	*GAPDH*	0.233
4	*RPL13A*	1.010	*GAPDH*	0.722	*GSK3B*	1.240	*YWHAZ*	0.268
5	*GAPDH*	1.207	*PPIA*	0.900	*PPIA*	1.492	*GSK3B*	0.449
6	*B2M*	1.378	*B2M*	1.031	*HPRT1*	1.558	*PPIA*	0.486
7	*HPRT1*	1.488	*HPRT1*	1.152	*GAPDH*	1.611	*HPRT1*	0.498
8	*PPIA*	1.516	*ACTB*	1.344	*B2M*	1.996	*B2M*	0.718
9	*ACTB*	2.273	-	-	*ACTB*	2.029	*ACTB*	0.813

**Table 4:** Sequences of RT-qPCR primers designed for the detection of potential reference genes and target genes in mice.

Gene Symbol	Gene Name	GenBank Accession Number	Primer Sequence (5′–3′) ^a^
*ACTB*	Actin, β	NM_007393.5	F: GTGCCCATCTACGAGGGCTATGCT
			R: TACCCAAGAAGGAAGGCTGGAAAA
*B2M*	β-2 microglobulin	NM_009735.3	F: AAGTATACTCACGCCACCCA
			R: AAGACCAGTCCTTGCTGAAG
*GAPDH*	Glyceraldehyde-3-phosphate dehydrogenase	NM_008084.3	F: AGTGCCAGCCTCGTCCCGTAG
			R: GTGCCGTTGAATTTGCCGTGAGTG
*GHR*	Growth hormone receptor	NM_001286370.1	F: CCAACTCGCCTCTACACCG
			R: GGGAAAGGACTACACCACCTG
*GSK3B*	Glycogen synthase kinase 3β	NM_019827.6	F: CCACCATCCTTATCCCTCCAC
			R: GTATCTGAGGCTGCTGTGGC
*HPRT1*	Hypoxanthine guanine phosphoribosyl transferase	NM_013556.2	F: CAGTCCCAGCGTCGTGATTA
			R: TCGAGCAAGTCTTTCAGTCCT
*IGF1*	Insulin-like growth factor 1	NM_010512.4	F: CCAAACACAATTCTCCTTCC
			R: GCTACAGCAACCTGTGATTG
*PPIA*	Peptidylprolyl isomerase A	NM_008907.1	F: GCGTCTCCTTGAGCTGTT
			R: AAGTCACCACCCTGGCAC
*RN18S*	18S ribosomal RNA	NR_003278.3	F: ACGGACAGGATTGACAGATT
			R: GCCAGAGTCTCGTTCGTTAT
*RPL13A*	Ribosomal protein L13A	NM_009438.5	F: TGACAAGAAAAAGCGGATGGTG
			R: GCTGTCACTGCCTGGTACTT
*YWHAZ*	Tyr 3-monooxygenase/Trp 5-monooxygenase activation protein, Z polypeptide	NM_011740.3	F: CCAGGACCTAAAAGGGTCGG
			R: ACACACCGAACTGTTGTCGT

^a^ F = forward primer, R = reverse primer.

**Table 5:** Properties of RT-qPCR primers designed for the detection of potential reference genes and target genes in mice.

Gene Symbol	Exon–Exon Junction ^a,b^	Tm (°C)	Product Length (bp)
*ACTB*	F: no	63	319
	R: yes	58	
*B2M*	F: no	56	162
	R: no	55	
*GAPDH*	F: yes	64	171
	R: no	61	
*GHR*	F: no	58	104
	R: no	57	
*GSK3B*	F: no	57	79
	R: yes	58	
*HPRT1*	F: yes	62	142
	R: yes	59	
*IGF1*	F: no	57	97
	R: no	57	
*PPIA*	F: no	55	145
	R: yes	58	
*RN18S*	F: no	57	118
	R: no	57	
*RPL13A*	F: yes	64	126
	R: no	58	
*YWHAZ*	F: no	63	115
	R: no	59	

^a^ F = forward primer, R = reverse primer; ^b^ Primers that span exon–exon junctions were selected whenever possible to avoid amplification of potentially remaining genomic DNA.

## Supplementary Materials

Supplementary materials can be found at www.mdpi.com/1422-0067/18/5/1060/s1. Figure S1. Melting curves of potential reference genes and target genes under study; Figure S2. Gel electrophoresis of RT-qPCR products; Figure S3. GSK3B protein content in skeletal muscle of growing mice overexpressing GH; Table S1. Raw *C*q values of potential reference genes determined for the same amount of total RNA; Table S2. Raw *C*q values of potential reference genes determined for the same amount of isolated mRNA; Table S3. Relative expression levels of potential reference genes determined for the same amount of total RNA; Table S4. Relative expression levels of potential reference genes determined for the same amount of isolated mRNA; Table S5. Normalization factor and relative expression levels of target genes; File S1. Additional discussion on the gene rankings obtained with algorithms.
