# Supplementary material for: Analysis of Different Approaches for the Selection of Reference Genes in RT-qPCR Experiments: A Case Study in Skeletal Muscle of Growing Mice
Source: Int J Mol Sci. 2017 May 16;18(5):1060. doi: 10.3390/ijms18051060 (PMC5454972; doi:10.3390/ijms18051060)

Figure S1: Melting curves of potential reference genes and target genes under study. A single peak detected in the melting curves examination confirmed the amplification of unique products by each primer pair and no primer dimer formation.

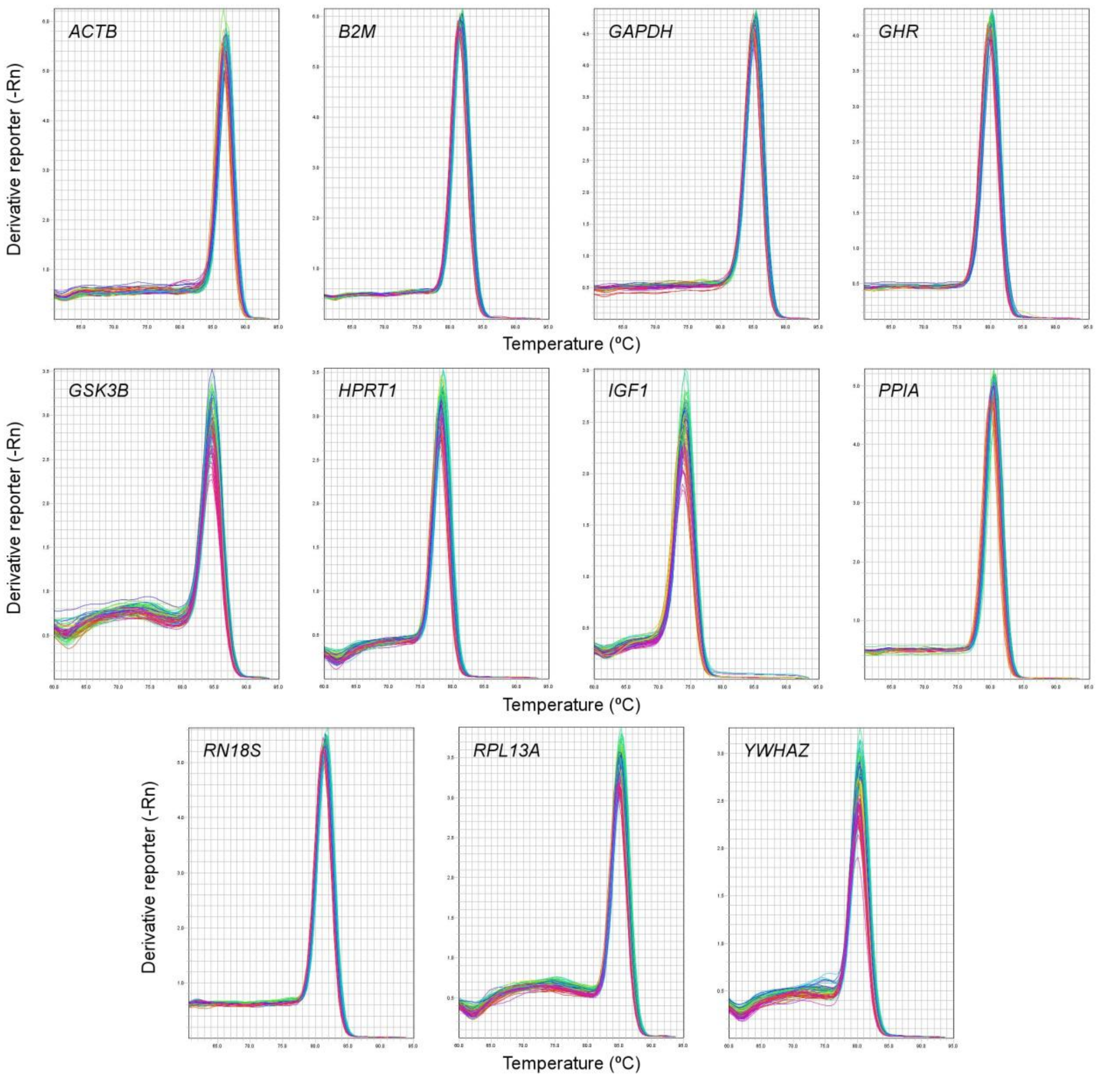

Supplement: Supplementary file 1 [file ijms-18-01060-s001.zip › ijms-191261-supplementary/Figure S1.pdf]
