# Supplementary material for: Analysis of Different Approaches for the Selection of Reference Genes in RT-qPCR Experiments: A Case Study in Skeletal Muscle of Growing Mice
Source: Int J Mol Sci. 2017 May 16;18(5):1060. doi: 10.3390/ijms18051060 (PMC5454972; doi:10.3390/ijms18051060)

Figure S2: Gel electrophoresis of RT-qPCR products. The banding pattern observed on a 2% agarose gel confirmed the amplification of unique products of expected size by each primer pair.

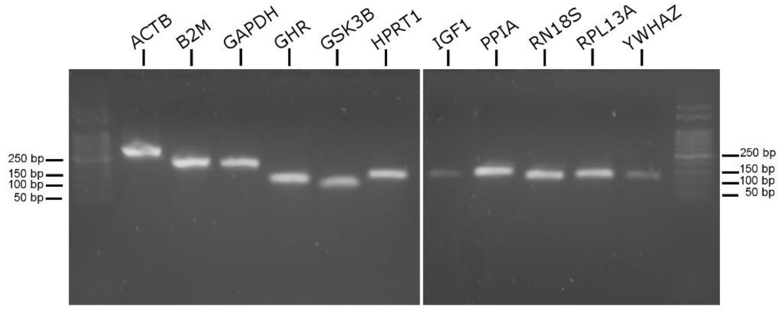

Supplement: Supplementary file 1 [file ijms-18-01060-s001.zip › ijms-191261-supplementary/Figure S2.pdf]
