# Supplementary material for: Analysis of Different Approaches for the Selection of Reference Genes in RT-qPCR Experiments: A Case Study in Skeletal Muscle of Growing Mice
Source: Int J Mol Sci. 2017 May 16;18(5):1060. doi: 10.3390/ijms18051060 (PMC5454972; doi:10.3390/ijms18051060)

Figure S3: GSK3B protein content in skeletal muscle of growing mice overexpressing GH. Equivalent amounts of skeletal muscle extracts from 2-, 4- and 9- week-old (2w, 4w, 9w) normal (N) and transgenic (T) mice were analyzed by western blotting with anti-GSK3B antibody. Loading control was performed with anti-ACTB antibody. Data are the mean ± *SEM* of the indicated *n* number of samples per group, each one representing a different animal. Normal distribution was confirmed by the D'Agostino-Pearson test before analysis by two-way ANOVA followed by the Bonferroni post-test. Different letters denote significant differences by age; small letters correspond to normal mice and capital letters to transgenic animals. A *p*-value lower than 0.05 was considered statistically significant. Representative immunoblots are shown.

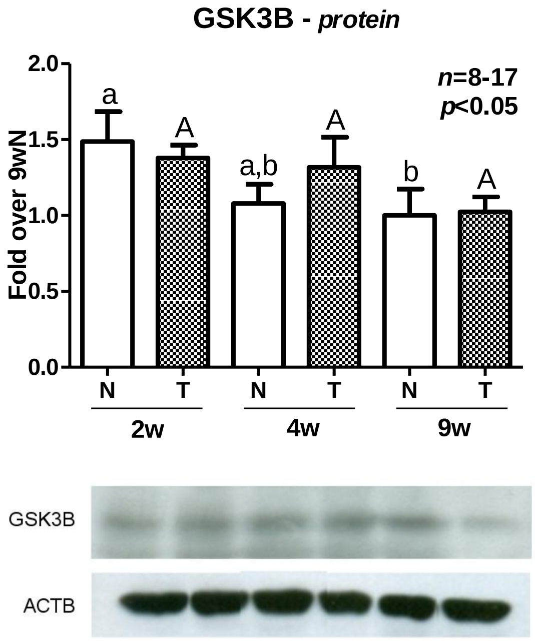

Supplement: Supplementary file 1 [file ijms-18-01060-s001.zip › ijms-191261-supplementary/Figure S3.pdf]
