# Supplementary material for: Analysis of Different Approaches for the Selection of Reference Genes in RT-qPCR Experiments: A Case Study in Skeletal Muscle of Growing Mice
Source: Int J Mol Sci. 2017 May 16;18(5):1060. doi: 10.3390/ijms18051060 (PMC5454972; doi:10.3390/ijms18051060)
Supplement: Supplementary file 1 [file ijms-18-01060-s001.zip › ijms-191261-supplementary/File S1.pdf]

Additional discussion on the gene rankings obtained with algorithms

Considering that geNorm and the Comparative ΔCq method evaluate the pairwise variation of a particular gene with all other genes, they tend to select those with the highest degree of similarity of expression profiles across the sample set as the most stable [1]. Consequently, *GSK3B*, that displayed a decreasing trend with age like the other genes, is ranked higher than *B2M* by these algorithms. These results highlight the fact that if only one stably expressed gene, i.e. a gene with an expression level that is not affected by experimental conditions, was to be present among a group of candidates, these algorithms would not recognize it as the most stably expressed, as it was previously reported [2]. As a consequence of their stability value definition, the outcome of these algorithms is easily affected by genes that behave all in the same way, like co-regulated genes. geNorm and the Comparative ΔCq method developers warned about this issue and they advised to choose genes that belong to different families and with different cellular functions in order to reduce the possibility of selecting co-regulated genes [3,4]. However, it is not always possible to assume that independency without any further testing. BestKeeper also recognized *GSK3B* as a more stably expressed gene than *B2M*, but this discrepancy was due to the higher dispersion of the results obtained for *B2M* that increases its standard deviation (*SD*) value, since the calculation of this stability value does not imply a comparison between genes. However, this algorithm depicted the lack of correlation between *B2M* and the BestKeeper index as it yielded an *R* value for the Pearson correlation test lower than 0.5, while the other genes led to an *R* value higher than 0.8.

In contrast to the results of the other algorithms, NormFinder found *GSK3B* and *B2M* more similar in terms of expression stability. However, it was not because it recognized both as more stably expressed due to their low intergroup variation, but as a consequence of *GSK3B* being ranked in a lowest position. While NormFinder placed it in the fifth position, despite this gene exhibiting not only a low deviation between ages but also a low intragroup variation, the rest of the algorithms ranked it among the top three genes regarding expression stability. A similar situation was reported by Kortner and coworkers who found that the gene with the lowest coefficient of variation in their study was the second lowest ranked by NormFinder, and also by geNorm, because it showed a low correlation with the expression pattern of the majority of the genes evaluated. These authors explained that NormFinder, although it assesses separately inter and intragroup variation, is also sensitive to groups of genes that behave all in the same way. That is because, instead of assuming that the individual candidate genes do not display systematic intergroup variation, it assumes that the average of all candidate genes display no systematic variation [5].

1. Andersen, C.L.; Jensen, J.L.; Orntoft, T.F. Normalization of real-time quantitative reverse transcription-PCR data: A model-based variance estimation approach to identify genes suited for normalization, applied to bladder and colon cancer data sets. *Cancer Res* **2004**, *64*, 5245-5250.
2. De Santis, C.; Smith-Keune, C.; Jerry, D.R. Normalizing RT-qPCR data: Are we getting the right answers? An appraisal of normalization approaches and internal reference genes from a case study in the finfish *Lates calcarifer*. *Mar Biotechnol (NY)* **2011**, *13*, 170-180.
3. Vandesompele, J.; De Preter, K.; Pattyn, F.; Poppe, B.; Van Roy, N.; De Paepe, A.; Speleman, F. Accurate normalization of real-time quantitative RT-PCR data by geometric averaging of multiple internal control genes. *Genome Biol* **2002**, *3*, research0034.0031–research0034.0011.
4. Silver, N.; Best, S.; Jiang, J.; Thein, S.L. Selection of housekeeping genes for gene expression studies in human reticulocytes using real-time PCR. *BMC Mol Biol* **2006**, *7*, 1-9.

5. Kortner, T.M.; Valen, E.C.; Kortner, H.; Marjara, I.S.; Krogdahl, A.; Bakke, A.M. Candidate reference genes for quantitative real-time PCR (qPCR) assays during development of a diet-related enteropathy in atlantic salmon (*Salmo salar l.*) and the potential pitfalls of uncritical use of normalization software tools. *Aquaculture* **2011**, *318*, 355-363.
