# Supplementary material for: Analysis of Different Approaches for the Selection of Reference Genes in RT-qPCR Experiments: A Case Study in Skeletal Muscle of Growing Mice
Source: Int J Mol Sci. 2017 May 16;18(5):1060. doi: 10.3390/ijms18051060 (PMC5454972; doi:10.3390/ijms18051060)
Supplement: Supplementary file 1 [file ijms-18-01060-s001.zip › ijms-191261-supplementary/S1 Table.pdf]

S1 Table. **Raw *Cq* values of potential reference genes determined for the same amount of total RNA.** Nine potential reference genes were analyzed by RT-qPCR in 51 skeletal muscle samples of 2-, 4- and 9-week-old normal and transgenic mice.

Set nº	Age[a]	Gender[b]	Genotype[c]	Mean Cq										
				ACTB	*B2M*	*GAPDH*	*GHR*	*GSK3B*	*HPRT1*	*IGF1*	*PPIA*	*RN18S*	*RPL13A*	*YWHAZ*
1	2w	F	N	23,401	24,189	22,369	26,586	24,308	26,156	25,624	24,182	11,151	20,439	30,546
		F	T	22,505	24,106	22,167	25,779	24,434	26,039	24,819	23,220	10,832	20,111	30,472
		M	N	22,140	24,075	22,266	25,449	24,518	26,013	25,489	22,977	10,785	20,702	30,345
		M	T	23,080	25,360	22,129	25,887	24,303	29,598	25,279	23,381	10,713	20,743	30,852
	4w	F	N	23,434	24,676	22,589	25,617	24,277	31,204	26,306	24,111	10,951	21,272	31,620
		F	T	23,651	23,529	23,773	26,026	24,766	28,489	25,958	23,951	11,717	21,164	30,808
		M	N	24,564	24,350	22,971	25,704	24,143	26,573	26,348	24,077	10,330	21,079	31,106
		M	T	23,460	23,501	24,345	26,211	25,027	28,314	26,859	24,675	11,433	21,309	31,918
	9w	F	N	29,305	24,956	25,693	29,134	25,535	31,245	29,102	28,167	13,344	22,806	32,226
		F	T	28,201	24,293	25,443	26,871	25,093	29,071	27,855	25,618	12,783	22,032	31,842
		M	N	30,860	24,252	25,078	27,572	25,150	30,806	29,214	26,887	13,414	23,000	31,835
		M	T	26,922	23,630	24,762	26,686	25,430	28,751	27,340	26,380	12,881	22,068	31,983
2	2w	F	N	22,849	25,306	22,685	26,307	24,040	26,105	25,707	22,990	11,514	20,358	31,027
		F	T	24,094	24,612	23,392	26,323	24,729	26,242	25,719	23,565	11,135	20,693	31,088
		M	N	23,879	24,651	21,801	27,752	24,994	26,572	26,375	24,377	10,857	20,747	31,561
		M	T	22,040	26,488	24,307	25,067	24,267	25,403	24,766	22,780	10,101	20,175	30,625
	4w	F	N	31,373	24,312	27,014	27,967	25,699	30,997	29,242	27,879	14,737	22,719	33,067
		F	T	23,734	23,389	23,256	25,878	24,032	25,807	25,188	23,749	11,200	20,716	31,061
		M	N	24,336	24,206	23,041	26,675	24,588	26,890	25,203	24,979	10,759	20,919	30,838
		M	T	26,216	23,316	25,321	27,932	25,507	28,255	27,908	25,603	12,750	21,166	31,646
	9w	F	N	26,614	24,296	23,292	27,183	25,159	27,461	28,856	24,956	12,853	21,920	31,452
		F	T	31,451	26,314	26,995	27,242	26,300	30,823	30,270	28,300	14,505	23,368	33,019
		M	N	23,616	22,735	22,700	25,057	24,276	26,148	26,672	23,602	11,018	21,282	31,626
		M	T	29,410	23,734	25,663	27,342	26,337	29,045	29,727	26,309	13,311	22,710	32,929
3	2w	F	N	24,470	24,457	24,472	27,196	25,689	26,940	26,951	24,932	12,178	21,413	31,368
		M	N	22,919	27,465	23,297	26,662	24,817	26,393	26,454	23,944	11,113	20,995	31,209
		M	T	23,911	25,808	24,454	27,404	25,395	26,879	26,472	24,552	11,794	21,403	31,732
	4w	F	N	24,768	27,409	24,019	27,273	25,495	28,375		26,852	12,283	23,330	32,196
		F	T	24,120	23,614	23,984	25,976	25,180	26,321	25,916	23,888	11,437	21,444	31,680
		M	N	23,588	23,359	24,346	26,373	25,156	26,929	26,996	24,469	11,927	21,493	31,704
		M	T	24,826	24,439	24,795	27,010	25,674	27,544	27,219	25,552	12,363	21,938	32,973
	9w	M	T	28,115	24,323	25,672	27,436	25,279	27,962	29,037	25,580	13,599	22,099	32,374
4	2w	F	T	23,832	24,208	23,495	26,092	24,572	25,967	25,919	23,166	11,533	20,621	30,908
		M	N	23,551	24,121	23,007	26,122	24,417	25,722	25,117	23,087	11,286	20,060	30,671
		M	T	22,210	24,566	22,599	25,315	23,922	25,345	24,680	22,910	10,823	20,250	30,222
	4w	F	N	27,436	23,799	24,208	29,153	24,329	26,148	26,382	26,667	11,740	20,735	31,407
		F	T	24,950	24,975	24,815	26,972	25,482	27,889	27,098	25,122	11,967	22,396	32,375
		M	N	25,292	24,761	23,850	27,320	25,480	28,236	27,171	28,995	11,588	21,973	32,452
		M	T	24,867	26,643	26,262	28,943	26,780	29,877	28,870	27,136	12,468	23,705	32,893
	9w	F	N	29,033	25,491	24,837	28,797	25,883	27,858	29,764	27,739	13,035	22,049	32,670
		F	T	26,074	26,365	25,310	27,456	25,991	28,333	28,672	26,366	12,528	22,502	32,911
5	2w	F	N	26,480	28,531	22,339	28,210	25,985	28,957	27,954	26,004	11,686	23,007	33,150
		M	N	25,668	28,379	22,090	27,519	25,433	29,881	27,124	25,457	11,478	22,990	33,278
		M	T	24,384	27,644	21,767	27,772	23,877	27,956	25,564	24,581	11,188	22,604	32,150
	4w	F	N	25,717	26,110	22,110	27,073	25,085	27,940	28,090	25,540	12,363	21,963	32,022
		F	T	26,080	27,213	22,909	27,047	25,084	27,779	26,596	24,627	12,969	21,837	31,963
		M	N	27,050	27,533	22,657	28,191	25,556	29,150	28,220	26,006	12,661	23,196	32,984
		M	T	26,194	25,768	22,092	27,245	25,343	28,557	26,584	25,182	12,178	22,833	32,314
	9w	F	T	28,400	27,916	25,996	30,028	27,038	31,171	30,451	27,511	15,544	24,272	33,946
		M	N	27,755	27,851	22,949	27,742	25,546	30,445	30,719	26,728	13,171	24,279	34,485
		M	T	25,058	25,613	25,121	28,281	25,658	28,976	28,809	25,913	14,752	22,190	32,216

	Mean Cq										
	ACTB	*B2M*	*GAPDH*	*GHR*	*GSK3B*	*HPRT1*	*IGF1*	*PPIA*	*RN18S*	*RPL13A*	*YWHAZ*
NTC[d]	Undetermined	Undetermined	Undetermined	Undetermined	36,344	Undetermined	Undetermined	Undetermined	34,775	37,704	Undetermined
NRTC[e]	Undetermined	Undetermined	Undetermined	Undetermined	34,152	Undetermined	Undetermined	36,450	32,944	37,953	Undetermined
mean	25,731	25,655	23,970	27,320	25,881	27,984	27,545	25,845	13,512	22,979	32,226
maximum	31,451	28,531	27,014	30,028	36,344	31,245	30,719	36,450	34,775	37,953	34,485
minimum	22,040	22,735	21,767	25,057	23,877	25,345	24,680	22,780	10,101	20,060	30,222
25% percentile	23,634	24,155	22,671	26,167	24,476	26,357	25,917	23,947	11,170	20,833	31,097
75% percentile	26,768	26,212	24,826	27,657	25,541	29,011	28,559	26,337	12,818	22,657	32,413

[a] 2w: 2-week-old, 4w: 4-week-old, 9w: 9-week-old

[b] F: female, M: male

[c] N: normal, T: bGH-transgenic

[d] NTC: non-template control

[e] NRTC: no-reverse transcription control
