# Supplementary material for: Analysis of Different Approaches for the Selection of Reference Genes in RT-qPCR Experiments: A Case Study in Skeletal Muscle of Growing Mice
Source: Int J Mol Sci. 2017 May 16;18(5):1060. doi: 10.3390/ijms18051060 (PMC5454972; doi:10.3390/ijms18051060)
Supplement: Supplementary file 1 [file ijms-18-01060-s001.zip › ijms-191261-supplementary/S2 Table.pdf]

S2 Table. **Raw *Cq* values of potential reference genes determined for the same amount of isolated mRNA.** Eight potential reference genes were analyzed by RT-qPCR in 17 skeletal muscle samples of 2-, 4- and 9-week-old normal mice.

Set nº	Age[a]	Gender[b]	Genotype[c]	Mean Cq							
				ACTB	*B2M*	*GAPDH*	*GSK3B*	*HPRT1*	*PPIA*	*RPL13A*	*YWHAZ*
1	2w	F	N	31,137	23,945	25,973	30,299	28,143	26,703	23,253	33,238
		M	N	25,934	22,594	23,056	27,012	26,062	24,495	21,181	31,481
	4w	F	N	26,484	23,942	22,812	27,265	26,003	24,724	21,398	32,050
		M	N	25,818	22,537	23,077	27,857	25,999	24,350	21,082	31,008
	9w	F	N	31,980	24,151	26,066	29,485	29,618	27,265	23,938	33,211
		M	N	30,698	23,916	25,927	29,673	28,978	26,765	22,937	33,221
2	2w	F	N	28,726	22,562	24,355	29,353	27,482	25,903	22,129	35,028
		M	N	30,196	22,960	25,551	29,411	28,842	27,049	22,980	34,906
	4w	F	N	28,264	22,783	23,807	27,490	27,286	25,839	21,903	33,335
	9w	F	N	29,509	23,606	25,854	30,374	28,691	27,035	23,574	35,736
		M	N	28,710	22,332	23,901	29,387	27,251	25,806	22,104	33,282
3	2w	F	N	31,303	23,576	25,622	30,124	28,415	27,119	22,968	34,425
		M	N	34,021	23,678	27,297	30,090	31,497	29,096	23,833	34,239
	4w	F	N	29,393	23,944	24,988	29,052	27,944	26,756	22,957	34,135
		M	N	30,585	24,043	25,918	29,141	29,335	27,967	23,159	34,428
	9w	F	N	30,242	24,049	25,342	29,191	28,663	27,516	23,351	34,621
		M	N	30,392	23,928	26,107	30,190	28,885	27,425	23,222	35,017
			mean	29,611	23,444	25,038	29,141	28,182	26,577	22,704	33,727
			maximum	34,021	24,151	27,297	30,374	31,497	29,096	23,938	35,736
			minimum	25,818	22,332	22,812	27,012	25,999	24,350	21,082	31,008
			NTC[d]	Undetermined	Undetermined	Undetermined	Undetermined	Undetermined	Undetermined	33,760	Undetermined
			NRTC[e]	Undetermined	Undetermined	Undetermined	Undetermined	Undetermined	Undetermined	32,875	Undetermined

[a] 2w: 2-week-old, 4w: 4-week-old, 9w: 9-week-old
[b] F: female, M: male
[c] N: normal
[d] NTC: non-template control
[e] NRTC: no-reverse transcription control
