# Supplementary material for: Analysis of Different Approaches for the Selection of Reference Genes in RT-qPCR Experiments: A Case Study in Skeletal Muscle of Growing Mice
Source: Int J Mol Sci. 2017 May 16;18(5):1060. doi: 10.3390/ijms18051060 (PMC5454972; doi:10.3390/ijms18051060)
Supplement: Supplementary file 1 [file ijms-18-01060-s001.zip › ijms-191261-supplementary/S3 Table.pdf]

S3 Table. **Relative expression levels of potential reference genes determined for the same amount of total RNA.** The mean ± *SEM* of the relative expression levels of reference genes for each experimental condition are shown.

Age[a]	Genotype[b]	Relative expression levels (Mean ± SEM)								
		ACTB	*B2M*	*GAPDH*	*GSK3B*	*HPRT1*	*PPIA*	*RN18S*	*RPL13A*	*YWHAZ*
2w	N	5,51 ± 1,32	0,65 ± 0,11	2,14 ± 0,36	1,24 ± 0,17	2,57 ± 0,38	2,89 ± 0,51	2,46 ± 0,22	2,64 ± 0,30	2,03 ± 0,24
	T	7,87 ± 1,81	0,58 ± 0,11	1,73 ± 0,36	1,55 ± 0,16	3,04 ± 0,59	3,71 ± 0,50	2,83 ± 0,39	2,82 ± 0,32	2,47 ± 0,23
4w	N	2,27 ± 0,59	0,98 ± 0,17	1,24 ± 0,21	1,21 ± 0,14	1,47 ± 0,35	1,19 ± 0,28	1,94 ± 0,36	1,79 ± 0,30	1,51 ± 0,25
	T	2,69 ± 0,52	1,33 ± 0,23	0,85 ± 0,14	1,02 ± 0,14	1,61 ± 0,41	1,89 ± 0,35	1,51 ± 0,18	1,83 ± 0,31	1,56 ± 0,30
9w	N	1,00 ± 0,77	1,00 ± 0,33	1,00 ± 0,31	1,00 ± 0,16	1,00 ± 0,47	1,00 ± 0,48	1,00 ± 0,33	1,00 ± 0,19	1,00 ± 0,16
	T	0,55 ± 0,27	0,79 ± 0,18	0,30 ± 0,05	0,68 ± 0,09	0,56 ± 0,13	0,66 ± 0,14	0,52 ± 0,09	1,11 ± 0,29	1,00 ± 0,26

[a] 2w: 2-week-old, 4w: 4-week-old, 9w: 9-week-old
[b] N: normal, T: bGH-transgenic
