# Supplementary material for: Analysis of Different Approaches for the Selection of Reference Genes in RT-qPCR Experiments: A Case Study in Skeletal Muscle of Growing Mice
Source: Int J Mol Sci. 2017 May 16;18(5):1060. doi: 10.3390/ijms18051060 (PMC5454972; doi:10.3390/ijms18051060)
Supplement: Supplementary file 1 [file ijms-18-01060-s001.zip › ijms-191261-supplementary/S4 Table.pdf]

S4 Table. **Relative expression levels of potential reference genes determined for the same amount of isolated mRNA.** The mean ± *SEM* of the relative expression levels of reference genes for each experimental condition are shown.

Age[a]	Genotype[b]	Relative expression levels (Mean ± SEM)							
		ACTB	*B2M*	*GAPDH*	*GSK3B*	*HPRT1*	*PPIA*	*RPL13A*	*YWHAZ*
2w	N	11,67 ± 5,12	1,34 ± 0,30	3,63 ± 1,26	2,26 ± 0,61	4,10 ± 1,34	4,51 ± 1,34	2,48 ± 0,70	5,37 ± 1,32
4w	N	2,95 ± 0,61	2,05 ± 0,24	2,30 ± 0,56	1,27 ± 0,38	2,25 ± 0,46	2,40 ± 0,41	1,77 ± 0,33	1,38 ± 0,38
9w	N	1,00 ± 0,30	1,00 ± 0,06	1,00 ± 0,19	1,00 ± 0,21	1,00 ± 0,20	1,00 ± 0,18	1,00 ± 0,08	1,00 ± 0,09

[a] 2w: 2-week-old, 4w: 4-week-old, 9w: 9-week-old
[b] N: normal
