# Supplementary material for: Analysis of Different Approaches for the Selection of Reference Genes in RT-qPCR Experiments: A Case Study in Skeletal Muscle of Growing Mice
Source: Int J Mol Sci. 2017 May 16;18(5):1060. doi: 10.3390/ijms18051060 (PMC5454972; doi:10.3390/ijms18051060)
Supplement: Supplementary file 1 [file ijms-18-01060-s001.zip › ijms-191261-supplementary/S5 Table.pdf]

S5 Table. **Normalization factor and relative expression levels of target genes.** The mean ± *SEM* of the relative expression levels of target genes for each experimental condition are shown as raw data and normalized by *RPL13A*, *YWHAZ*, *GSK3B* and *RN18S* expression levels.

| Age[a] | Genotype[b] | Relative expression levels (Mean ± SEM) | | | | |
|---|---|---|---|---|---|---|
| | | NF[c] | *IGF1* raw data | *IGF1* norm.[d] | *GHR* raw data | *GHR* norm.[d] |
| 2w | N | 2,06 ± 0,17 | 5,75 ± 0,98 | 3,25 ± 0,31 | 1,97 ± 0,30 | 0,68 ± 0,07 |
| | T | 2,40 ± 0,22 | 10,65 ± 1,40 | 5,06 ± 0,28 | 2,88 ± 0,46 | 0,80 ± 0,08 |
| 4w | N | 1,59 ± 0,22 | 3,94 ± 0,93 | 2,68 ± 0,40 | 1,83 ± 0,35 | 0,87 ± 0,11 |
| | T | 1,45 ± 0,18 | 4,46 ± 0,94 | 3,46 ± 0,42 | 2,00 ± 0,29 | 0,97 ± 0,09 |
| 9w | N | 1,00 ± 0,18 | 1,00 ± 0,51 | 1,00 ± 0,27 | 1,00 ± 0,28 | 1,00 ± 0,24 |
| | T | 0,77 ± 0,11 | 0,92 ± 0,23 | 1,37 ± 0,24 | 1,20 ± 0,14 | 1,22 ± 0,22 |

[a] 2w: 2-week-old, 4w: 4-week-old, 9w: 9-week-old

[b] N: normal, T: bGH-transgenic

[c] NF: normalization factor calculated as the geometric mean of the relative expression levels of *RPL13A* , *YWHAZ* , *GSK3B* and *RN18S* for each sample

[d] norm: normalized by NF
